# Coronary Artery Calcium Score as a Graded Decision Tool

**DOI:** 10.1016/j.jacadv.2023.100664

**Published:** 2023-10-24

**Authors:** Sean Paul Gaine, Roger S. Blumenthal, Garima Sharma

**Affiliations:** a Division of Cardiology, Department of Medicine, Ciccarone Center for the Prevention of Cardiovascular Disease, Johns Hopkins University School of Medicine, Baltimore, Maryland, USA; b Inova Schar Heart and Vascular, Inova Health System, Falls Church, Virginia, USA

**Keywords:** atherosclerosis, coronary calcium score, primary prevention

Over the last 3 decades, coronary artery calcium (CAC) has emerged as a highly specific marker for coronary atherosclerosis. Agatston et al^1^ first described noncontrast-enhanced, electrocardiographically gated computed tomography as an effective tool to quantify CAC in 1990 and improve cardiovascular risk assessment. Since then, CAC has been studied extensively in myriad of population-based studies and has been shown to effectively stratify cardiovascular risk across ethnicities, irrespective of age, sex, and risk factor burden.^2^, ^3^, ^4^, ^5^, ^6^ Beyond risk stratification, CAC can identify high-risk patient subgroups who are more likely to benefit from more intensive primary prevention strategies. Quantification of CAC, distribution, location, and its association with high-risk plaque have added to our understanding of this innovative and yet simple decision tool.

The 2018 American College of Cardiology/American Heart Association (AHA)/Multisociety cholesterol guideline recommends the selective use of CAC scoring in primary prevention to aid in the decision-making process regarding statin therapy when there is uncertainty on the part of the clinician or patient.^7^ If the CAC score is >100 AU or ≥75th percentile of the CAC score distribution for a particular age/gender, initiating statin therapy is encouraged because of the predicted strong net benefit. However, therapeutic inertia remains an issue in implementing novel preventive strategies in those with high CAC scores but without atherosclerotic cardiovascular disease (ASCVD) events.^8^

Several studies have aimed to determine the appropriate intensity of treatment for individuals with elevated CAC scores who have not experienced ASCVD events when compared to those who have survived an ASCVD event. In other words, at what level of CAC do adults in the primary prevention setting have a risk comparable to those with prior ASCVD events, and thus warrant more aggressive risk factor reduction (Figure 1).

**Figure 1:**
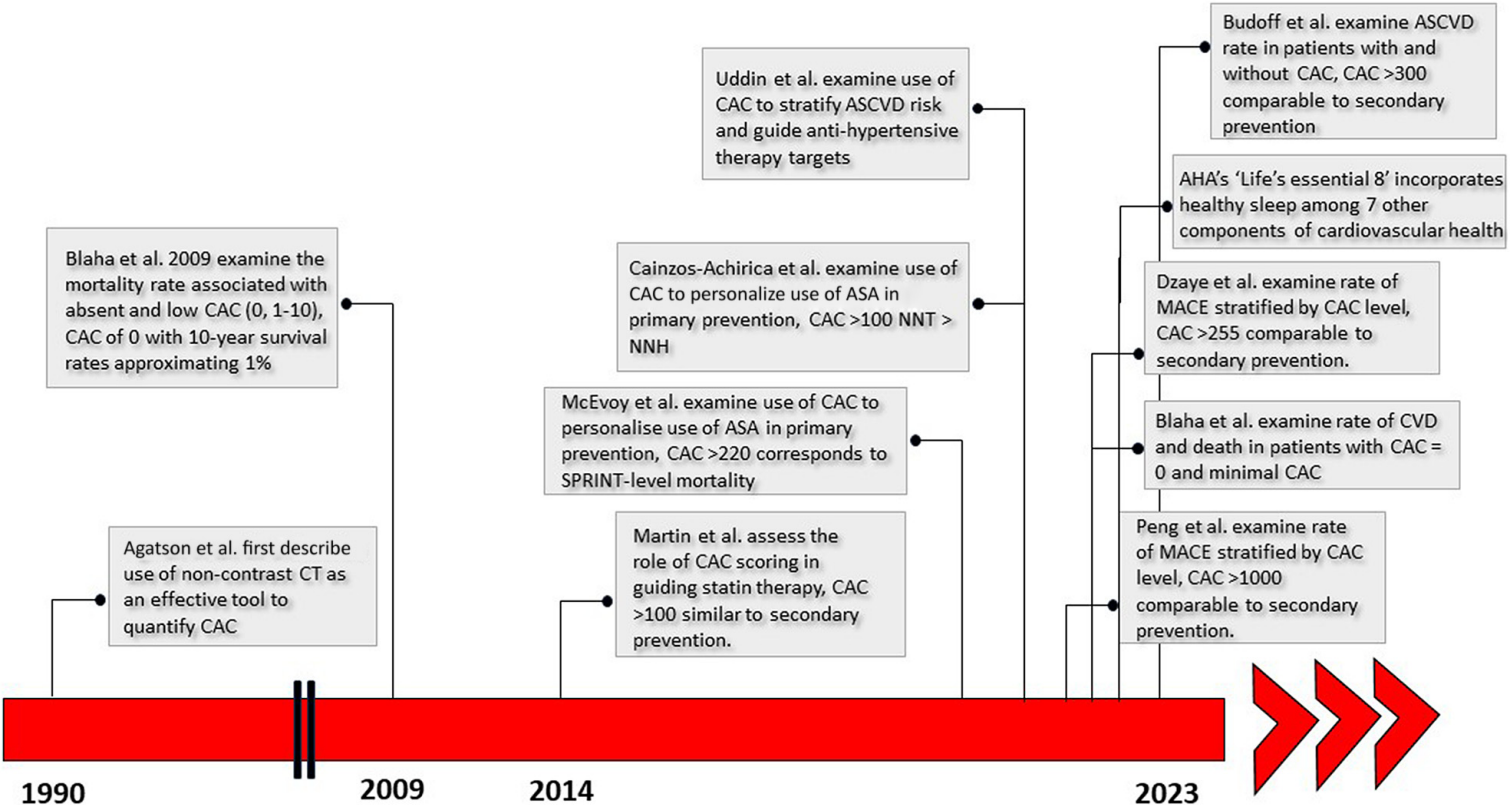
**Important Studies Examining the Use of Coronary Artery Calcium Scoring for ASCVD Risk Stratification** AHA = American Heart Association; ASA = aspirin; ASCVD = atherosclerotic cardiovascular disease; CT = computed tomography; CVD = cardiovascular disease; MACE = major adverse cardiovascular events; NNH = number needed to harm; NNT = number needed to treat.

In 2014, Martin et al^9^ assessed the role of CAC scoring in guiding statin therapy allocation based on absolute cardiovascular disease (CVD) risk in 5,534 participants from the Multi-Ethnic Study of Atherosclerosis (MESA) study who were not taking dyslipidemia medications at baseline. Participants were categorized based on their CAC scores (>0 or ≥100) and the presence of lipid abnormalities and the main outcome measure was incident CVD events. Over a median follow-up of 7.6 years, individuals with CAC ≥100 had CVD rates similar to secondary prevention populations across the spectrum of dyslipidemia, suggesting that CAC scoring can align statin therapy with absolute CVD risk.

Subsequently, Peng et al^10^ examined the rate of major adverse cardiovascular events (MACE) seen in patients with CAC scores of 0, 1 to 99, 100 to 399, 400 to 999, and ≥1,000 over a mean follow-up period of 13.6 years. Those with CAC ≥1,000 had an event rate similar to the average stable treated secondary prevention patient in the FOURIER (Evolocumab and Clinical Outcomes in Patients with Cardiovascular Disease) trial.^11^ Another study by Dzaye et al analyzed patients from the CAC Consortium who were over 50 years old with a 10-year ASCVD risk >7.5% (n = 20,207). The annualized ASCVD mortality rate among FOURIER participants corresponded to a CAC score of 781 (418-1,467) and a CAC score of 255 (162-394) corresponded to an ASCVD mortality rate equivalent to the lowest risk FOURIER subgroup.^12^

Most recently, Budoff et al^13^ compared ASCVD event rates in individuals without a history of myocardial infarction or revascularization (categorized based on CAC scores) to event rates in those with established ASCVD. The study included 4,511 individuals from the CONFIRM (COronary CT Angiography EvaluatioN For Clinical Outcomes: An InteRnational Multicenter) registry without known coronary artery disease and 438 individuals with established ASCVD. Patients were followed for a median of 4.7 years, age (66 vs 63 years) and baseline prevalence of CVD risk factors were similar among those with CAC >300 and those with established ASCVD. Individuals with CAC scores >300 had similar rates of MACE (20%) and its components as those with established ASCVD (23%), correlating to an event rate of 73.9 and 77.8 per 1,000 person-years.

Conversely, a CAC score of 0 has been shown to confer a very low risk of future ASCVD events and mortality over 10 to 15 years, dubbed the ‘power of zero.’ Mitchell et al evaluated the impact of statin therapy in 13,644 patients followed for a median period of 9.4 years. They found that statin therapy was associated with reduced risk of MACE in patients with CAC but not in patients without CAC. The number needed to treat (NNT) was 3,571 in individuals with a CAC score of 0 to prevent first occurrence of MACE over 10 years compared with a NNT of 12 for CAC score >100.^14^ Similarly, in 2020, Blaha et al evaluated 66,363 asymptomatic patients from the CAC Consortium who underwent CAC scoring for clinical risk assessment. Over a mean follow-up period of 12 years, individuals with a CAC score of 0 had low rates of CVD mortality and all-cause death. Even those with a minimal CAC score of 1 to 10 had a 1.4-fold increased risk of death from any cause as compared with a CAC score of 0.^5^

A key lesson from the CAC Consortium was the appreciation for the prognostic value implied by a CAC score of 0. These participants were identified as a population group with favorable all-cause prognosis and stable low 12-year rates of CVD mortality. Thus, CAC scoring can be used as an important decision aid in de-risking patients. Based on the mounting evidence favoring CAC as a risk stratification tool, and particularly a CAC score of 0 as a negative risk factor, Greenland et al^15^ have distilled the current knowledge into an approach for selective use of CAC scoring to guide shared decision-making in the primary prevention of CVD.

Additionally, several studies have looked at the use of CAC to guide aspirin and antihypertensive therapy in primary prevention. Cainzos-Achirica et al^16^ examined 6,470 participants from the MESA registry and determined that among aspirin-naive individuals <70 years old without high bleeding risk, the NNT with aspirin to prevent 1 CVD event was higher or similar to the number needed to harm across different CVD risk categories. However, subgroups with higher CAC scores of >100 (especially >400) showed a much lower NNT compared to number needed to harm, indicating probable net benefit from aspirin use. The authors concluded that CAC scoring could provide improved risk assessment compared to the pooled cohort equations when guiding aspirin allocation in primary prevention.

In 2019, Uddin et al^17^ evaluated the use of CAC to stratify ASCVD risk in 6,375 hypertensive adults with SPRINT (Systolic Blood Pressure Intervention Trial)-level CVD mortality risk (0.43%/y) to identify which patients would benefit from aggressive blood pressure therapy; a CAC score of >220 corresponded to “SPRINT-level” risk of CVD mortality. Similarly, McEvoy et al^18^ evaluated the role of CAC in guiding the allocation of antihypertensive treatment in 3,733 participants from the MESA cohort with systolic blood pressure (SBP) ranging from 120 to 179 mm Hg. These patients were categorized based on SBP and estimated 10-year ASCVD risk and the risk of ASCVD or heart failure events based on CAC levels (0, 1-100, or >100) within each SBP and ASCVD risk subgroup. CAC scoring stratified the risk of events in individuals with SBP <160 mm Hg. The estimated NNT for a SBP goal of 120 mm Hg vs 135 to 140 mmHg varied significantly based on CAC levels when the predicted ASCVD risk was <15% and the baseline SBP was <160 mm Hg. These findings show that combining CAC imaging with ASCVD risk assessment may aid in determining personalized SBP goals.^18^

These studies provide substantial evidence for the use of CAC as a decision tool to stratify ASCVD risk and identify patients who would benefit from a more aggressive approach to treatment. While the absolute CAC value at which patients are at secondary prevention level risk may vary based on factors such as age, comorbidities, and population studied, the cumulative evidence strongly suggests that patients with CAC >100 and certainly >300 should be treated more aggressively, akin to the secondary prevention population.

The question remains as to how we can provide innovative care models aimed at personalized risk stratification. We propose a strategy which utilizes CAC to identify those at greatest risk of ASCVD and guide treatment intensity, as defined by 3 CAC groups: those with CAC >300, CAC >100, and CAC 0. There are several important areas of intervention.

1) Treating to lower LDL-cholesterol targets. Both the European Society of Cardiology and the 2022 American College of Cardiology Expert Consensus Statement recommend lower low-density lipoprotein cholesterol (LDL-C) targets (<55 mg/dL) in patients with established ASCVD at highest risk for recurrent events. Statin therapy remains the first-line treatment for effective LDL-C lowering with multiple additional options available including PCSK9 inhibitors, bempedoic acid, ezetimibe, and inclisiran. Those with CAC score >300 are at secondary prevention level of risk and should be treated aggressively to LDL-C targets of <55 mg/dL. Ezetimibe and/or bempedoic acid may be warranted in those not achieving these targets with statin monotherapy. For those with CAC >100, statin therapy should be strongly recommended with a target LDL-C of <70 mg/dl. In those with CAC 0, lower dose statin therapy can still be considered based on shared decision-making; however, an approach which focuses on lifestyle, diet, and exercise measures is reasonable.
2) Aspirin use. Patients with CAC >300 and low bleeding risk should be treated with aspirin for primary prevention. In those with CAC >100 and low bleeding risk, aspirin should be considered. In those with no or mild CAC, aspirin should probably not be used for primary prevention.
3) Better glycemic control and blood pressure control. Patients with CAC >300 (and possibly >100) and elevated SBP should be more aggressively treated for a SBP in the range of 120. Stronger consideration should also be given to adding an sodium-glucose cotransporter-2 inhibitor or glucagon-like peptide-1 receptor agonists in these higher risk patients.^19^^,^^20^
4) Diet, body mass index, and exercise. Patients with and without CAC benefit from lifestyle, dietary, and exercise interventions and this should be addressed at each visit. For those with CAC of 0, a treatment strategy based on these interventions alone is reasonable. When compared with individuals with normal body mass index, patients with CAC >300 are at greatly elevated risk of coronary heart disease, CVD, and all-cause mortality highlighting the importance of at least consideration of more aggressive weight loss measures in this population. While surgical approach is the only known intervention to reduce CVD risk, there are ongoing trials (SELECT and SURMOUNT-MMO) evaluating the effects of glucagon-like peptide 1 receptor agonist medications on CVD risk and mortality.
5) Sleep. Sleep irregularity has been linked to incident CVD. Patients with more irregular sleep timing (>120 minutes in a week) are more likely to have high CAC burden even after adjusting for CVD risk factors, obstructive sleep apnea, and sleep duration.^21^ Encouraging a regular sleep schedule and reducing variability in sleep may be an important modifiable risk factor to reduce CVD risk.

A patient with a CAC score of >300 has comparable ASCVD risk to that of a post myocardial infarction survivor or someone who had undergone arterial revascularization and should be treated to secondary prevention level LDL-C targets. The new data from Budoff et al for patients with CAC >300 represent a call to action to reverse the therapeutic inertia surrounding these patients. We propose a strategy which uses CAC score to guide treatment approach and degree of intensity in 3 groups (CAC >300, >100, 0). Using this model will help to intensify our approach, particularly in the high-risk (but event free to date) group of patients with CAC >300.

The story of CAC has been one of innovation and reorganization of guidelines, moving from specific groups to provide a more effective strategy to clinicians and patients in refining ASCVD risk. It continues to provide information to patients and clinicians as a strong and evidence-based marker for future ASCVD events and provides an additional tool to assess and optimize cardiovascular health.

Recently, the AHA revised and updated its definition of cardiovascular health (CVH) to include sleep hygiene as an additional measure to assess an individual’s overall health. The AHA’s ‘Life’s Essential 8’ now incorporates healthy sleep among 7 other components of cardiovascular health: healthy diet, participation in physical activity, avoidance of nicotine, healthy weight, healthy levels of blood lipids, blood glucose, and blood pressure.^8^

More clinicians may offer CAC scoring in those with suboptimal CVH and no prior ASCVD events for better optimization of treatment strategies if the patient has not made significant progress with optimizing their risk factor control. With these newer data on the prognostic significance of CAC >300, clinicians have yet another tool to drive clinical measure of CVH to optimum control. It may help us do what something what we already do better—incorporate the ABCDE’s and strongly consider treating such higher risk “primary prevention” patients with Aspirin, aim for a systolic Blood pressure in the range of 120 mmHg, aim for an LDL-Cholesterol preferably <55 mg/dL, counsel patients on optimizing Dietary and Exercise habits, and consider more aggressive Diabetes medications to improve glycemic control, focus on better sleep hygiene, achieving healthy body mass index and smoking cessation.

## Funding support and author disclosures

The authors have reported that they have no relationships relevant to the contents of this paper to disclose.
